# A Decade of Advancements: A Systematic Review of Effectiveness of Interventions to Reduce Burnout AmongMental Health Nurses

**DOI:** 10.3390/healthcare13172113

**Published:** 2025-08-25

**Authors:** Mark Fredrick Abundo, Adem Sav

**Affiliations:** School of Public Health and Social Work, Faculty of Health, Queensland University of Technology, Kelvin Grove, Brisbane, QLD 4059, Australia; markfredrick.abundo@connect.qut.edu.au

**Keywords:** burnout, mental health nurses, interventions, systematic review

## Abstract

**Background**: Burnout is a prevalent issue among mental health nurses. While various interventions have been implemented to address burnout, their effectiveness and sustainability remain unclear in specialised mental health settings. This systematic review aims to clearly evaluate the effectiveness of interventions specifically designed to reduce burnout among mental health nurses, focusing on intervention types, their impact, and the sustainability of results. **Methods**: A comprehensive search of databases (Embase, CINAHL, Medline, PubMed, Scopus, and Web of Science) identified studies on burnout reduction interventions for mental health nurses. Inclusion criteria focused on mental health nursing populations with pre- and post-intervention burnout measures. Methodological quality was assessed using JBI Critical Appraisal Tools. A narrative synthesis guideline was used to analyse data. **Results**: Among 2502 studies retrieved, only 4 met the inclusion criteria after a rigorous screening process. These studies explored specific intervention types, including a two-day burnout prevention workshop, an eight-week group-based psychoeducational programme, a twelve-week mindfulness-based psychoeducational intervention, and an eight-week guided self-help mindfulness programme delivered via a digital platform. Significant reductions in burnout were observed across these studies; however, the sustainability of these effects varied. Interventions of greater duration, such as the 12-week mindfulness-based programme and the 8-week group psychoeducational intervention, yielded more enduring improvements. In contrast, shorter interventions, like a two-day workshop, showed transient benefits that diminished over time. **Conclusions**: This review highlights a critical gap in research on burnout interventions for mental health nurses. While the reviewed interventions showed promise in reducing burnout, the findings underscore the need for sustainable, adaptable interventions and more robust research.

## 1. Introduction

Burnout is a chronic response to unmanaged workplace stress, marked by emotional exhaustion, detachment from work, and a reduced sense of accomplishment [1]. It can be caused by a variety of factors, including excessive workload, prolonged work hours, lack of control over job-related decisions, insufficient rewards for the effort put in, and inadequate support from colleagues and supervisors [2]. The effects of burnout are extensive, leading to decreased job performance, increased absenteeism, and a higher turnover rate [3]. Burnout also has significant implications for mental and physical health, contributing to conditions such as depression, anxiety, and cardiovascular diseases [4,5].

The global burden of burnout is substantial, affecting individuals across various professions and sectors [6]. For instance, in the United States of America (USA), Gerding et al. [7] estimated that workplace stress, including burnout, costs the economy over USD 300 billion annually due to absenteeism, reduced productivity, and healthcare expenses. In Japan, “karoshi”, or death from overwork, has led to government interventions to address the severe impacts of burnout on employees’ health and well-being [8]. Similarly, in the United Kingdom, burnout in the National Health Service (NHS) has been linked to high staff turnover and significant financial costs associated with recruiting and training new healthcare workers [9]. These accounts substantiate the World Health Organisation’s (WHO) recognition of burnout as an occupational phenomenon as listed in its International Classification of Diseases (ICD-11), underscoring its international relevance and impact [10].

In the healthcare sector, nurses are particularly vulnerable to burnout due to the demanding nature of their work [11]. A meta-analysis by Li et al. [12] found that 40.5% of emergency nurses reported experiencing high levels of emotional exhaustion, a key component of burnout. Although burnout is a significant concern across all nursing disciplines, mental health nurses face additional, unique stressors that make them particularly susceptible [13]. Mental health nurses operate in uniquely demanding environments that can differ substantially from general nursing contexts [14]. They are frequently exposed to emotionally intense situations, including managing patients with severe psychiatric disorders, behavioural crises, and long-term therapeutic relationships [15]. These conditions contribute to a heightened risk of emotional exhaustion, compassion fatigue, and burnout [16]. Mental health nurses often work in settings where verbal and physical aggression can be common, and where emotional labour is a central component of care [15]. These distinct stressors justify the need for targeted interventions and a focused review.

In response, various interventions have been introduced to address burnout in mental health nurses, ranging from resilience-building programmes to mindfulness and stress management techniques [13,17,18]. While these strategies have shown promise, the most effective methods for sustainably reducing burnout in this population remain unclear. We do not know which interventions effectively reduce burnout among mental health nurses under specific circumstances. Therefore, this systematic review aims to clearly evaluate the effectiveness of interventions specifically designed to reduce burnout among mental health nurses, focusing on intervention types, their impact, and the sustainability of results.

## 2. Materials and Methods

A systematic review was chosen for its ability to critically appraise, synthesise, and consolidate findings from multiple studies into a comprehensive summary [19]. The PICO (Participants, Interventions, Comparators, Outcomes) framework (see Table A1) provided a clear and specific research question, ensuring that the review is focused and methodologically sound. Regular consultations with the research team were held throughout the review process to ensure adherence to rigorous methodology and provide guidance on methodological decisions.

The PRISMA statement framework used in conjunction with Joanna Briggs Institute (JBI) reporting standards as reporting guidelines to enhance the completeness and transparency of this review. The PRISMA checklist and flow diagram were employed to systematically document the inclusion and exclusion of studies, from the initial search through to the final synthesis, thereby minimising research ‘waste’ and ensuring high-quality reporting [20].

To further enhance the rigour of the review, JBI methodology for systematic reviews of effectiveness (as outlined in the JBI Manual for Evidence Synthesis, 2024) was followed [21]. The execution of the review, including study selection, data extraction, and critical appraisal, was conducted using JBI tools and checklists. This ensures that the review process meets high standards of reliability and validity, strengthening the credibility of the findings. In line with this methodology, the review includes clearly defined inclusion and exclusion criteria, systematic literature searching, critical appraisal of the selected studies, and a detailed synthesis of findings.

A meta-analysis was not conducted due to both the limited number of studies found and the heterogeneity across them, with variations in intervention types, duration, and outcome measures making it unfeasible to combine data quantitatively [22]. Instead, a narrative synthesis approach following the Synthesis Without Meta-analysis (SWiM) guidelines by Campbell et al. [23] was followed.

### Search Strategy

A literature search was conducted across six major databases: Embase, CINAHL via EBSCOhost, Medline via EBSCOhost, PubMed, Scopus, and Web of Science. The following keywords were searched: (a) mental health nurses: psychiatric nurse “OR” mental health nurse “OR” community health nurse * “OR” behavioral health nurse “OR” behavioural health nurse “OR” psychiatric registered nurse “OR” psychiatric-mental health nurse “OR” community mental health nurse “OR” community psychiatric nurse *; (b) burnout: “burnout” “OR” occupational burnout “OR” staff burnout “OR” professional burnout “OR” career burnout “OR” psychological burnout “OR” psychological fatigue “OR” emotional exhaustion “OR” emotional fatigue”), (c) and interventions: “intervention * “OR” management “OR” program * “OR” strateg* “OR” workshop * “OR” training * “OR” seminar * “OR” wellness initiative *”. Citation chaining was also performed by reviewing the reference lists of included studies.

All records were exported to EndNote 20 and duplicates were removed. To be included, studies needed to focus on mental health nurses globally and workplace interventions aimed at reducing burnout among these nurses. Eligible designs included both quantitative and qualitative or mixed-methods studies and designs that involved RCTs, quasi-experimental, observational, and mixed-methods studies with pre- and post-intervention burnout measures. Studies that did not isolate data for mental health nurses did not measure burnout outcomes using validated scales, or were qualitative-only, reviews, or grey literature. Only studies published in English within the last 10 years and peer-reviewed in scientific journals were considered. Finally, disagreements regarding some papers were discussed and resolved by the two authors (see Table A2 for a summary of the inclusion and exclusion criteria).

In accordance with JBI methodology, studies were compared based on intervention type, setting, and outcomes, highlighting patterns in intervention duration and participant engagement. A predefined data extraction table was used to ensure consistency in capturing the critical aspects of each study, allowing for an organised comparison across the studies. The columns in the data extraction table were specifically designed to cover essential details, providing a structured format to categorise and analyse the studies.

## 3. Results

**Figure 1:**
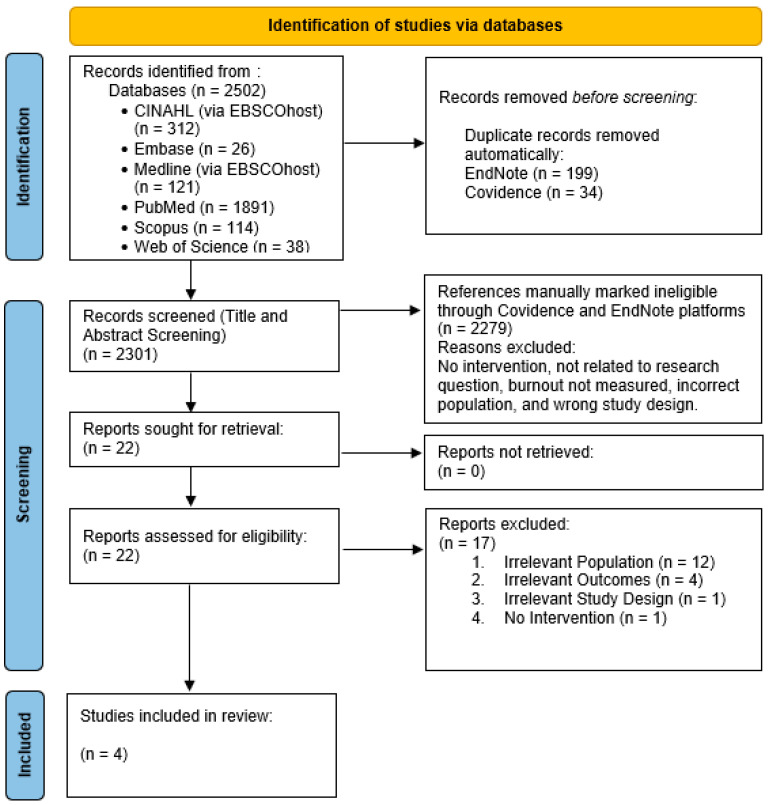
PRISMA 2020 flow diagram for a decade of advancements: a systematic review of interventions to reduce burnout among mental health nurses.

The search strategy identified 2502 records across six databases. Ultimately, four studies met the inclusion criteria (see Figure 1). The quality of the studies was assessed using the Cochrane RoB 2 tool for the randomised controlled trial (RCT) by Laker et al. [24] and Wang et al. [25] and the ROBINS-I (Risk Of Bias In Non-randomised Studies of Interventions) tool for the non-randomised studies by Alenezi et al. [26] and Wampole and Bressi [27] (see Table A3).

The two RCTs in the review provided relatively strong evidence. However, Wang et al. [25] raised concerns about deviations from intended interventions and outcome reporting. The risk of bias influenced the weight of each study’s findings in the final synthesis. Wampole and Bressi [27] had a higher risk of bias due to confounding and a small sample size, warranting caution in interpreting its findings. In contrast, Alenezi et al. [26] had moderate bias, with more reliable results due to stronger intervention classification and outcome measurement.

JBI Critical Appraisal Tools were used (see Table A4) for a thorough evaluation of study designs and bias. The RCTs by Laker et al. [24] and Wang et al. [25] received high ratings for strong randomization, large sample sizes, and validated measures, showing sustained reductions in burnout and significant reductions via a mobile platform, respectively. In contrast, Alenezi et al. [26] and Wampole and Bressi [27] faced methodological challenges leading to moderate-to-low ratings. Alenezi et al. [26] reported burnout reduction but diminishing effects over time, while Wampole & Bressi [27] showed limited generalizability and slight improvements.

### Characteristics of Included Studies

The four studies [24,25,26,27] included in this systematic review explored various interventions to reduce burnout among mental health nurses, conducted in different countries, including Saudi Arabia [26], the USA [27], the United Kingdom (UK) [24], and China [25]. These studies provided insights into global approaches to addressing burnout in this high-risk population. Despite geographical differences, all focused on mental health nurses working in high-stress environments and employed validated burnout measures like the Maslach Burnout Inventory (MBI), Oldenburg Burnout Inventory (OLBI) and the Connor–Davidson Resilience Scale (CD-RISC).

Alenezi et al. [26] conducted a quasi-experimental study using a non-equivalent pre-test and post-test design in Saudi Arabia with the largest sample size of 296 participants (154 in the intervention group and 142 in the control group). In contrast, Wampole and Bressi [27], a pre–post intervention pilot study from the USA, had the smallest sample size (*n* = 5). Laker et al. [24] conducted a pragmatic randomised controlled trial (pRCT) involving 173 participants, divided into two groups: an immediate intervention group and a delayed intervention group. Finally, Wang et al. [25], a randomised controlled trial (RCT) from China, involved 118 participants, divided into an intervention group (*n* = 52) and a control group (*n* = 47).

The studies employed various interventions to reduce burnout among mental health nurses. Alenezi et al. [26] used a two-day burnout prevention workshop, while Wampole and Bressi [27] applied a 12-week mindfulness-based psychoeducational intervention. Laker et al. (2023) [24] implemented an eight-week group-based psychoeducational intervention, like the one used by Wampole and Bressi [27] but with a larger sample size and different group structures. Wang et al. [25] delivered an eight-week guided self-help mindfulness-based intervention using audio and text materials via WeChat.

The studies [24,25,26,27] consistently noted that the setting itself, characterised by challenging patient interactions, high emotional demands, and often understaffed environments, played a significant role in the prevalence and intensity of burnout among mental health nurses. All studies employed validated measures to assess burnout, with the Maslach Burnout Inventory (MBI) being the most frequently used. In addition, other psychological outcomes were evaluated, including well-being [24], psychological resilience [25], and mindfulness [25]. Significant reductions in burnout were observed across the studies; for instance, Alenezi et al. [26] reported a reduction in burnout scores one month after the intervention but a partial return to baseline by six months. A summary of the key results for each study is presented in Table 1.

Alenezi et al.’s [26] quasi-experimental study, using a non-equivalent pre-test and post-test design, was conducted in a Saudi Arabian mental health hospital and involved 296 participants. The intervention group (*n* = 154) participated in a two-day (6 h per day) burnout prevention workshop, while the control group (*n* = 142) did not receive any intervention. The study observed a significant reduction in burnout scores one month after the workshop, as measured by MBI. However, the study found that burnout scores had partially returned to baseline by the six-month follow-up.

Wampole and Bressi’s [27] quasi-experimental pilot study (pre–post intervention) was conducted in an inpatient psychiatric facility in the USA with a sample size of only five participants. The intervention consisted of a 12-week mindfulness-based psychoeducational line, delivered for one hour each week. The study yielded mixed results: while there was a slight decrease in depersonalisation scores (MBI), emotional exhaustion increased slightly, and personal accomplishment remained unchanged.

Laker et al.’s [24] pRCT was conducted in the UK and involved 173 mental health nurses, who were divided into an immediate intervention group (*n* = 83) and a delayed intervention group (*n* = 90). The intervention was an eight-week group-based psychoeducational programme, with weekly 90 min sessions. The study found moderate reductions in burnout (as measured by the OLBI) and improvements in well-being (assessed via the Warwick–Edinburgh Mental Well-being Scale (WEMWBS)). These effects were sustained over time, with some reductions in effect size by the six-month follow-up.

Finally, Wang et al.’s [25] RCT, conducted in China, included 118 psychiatric nurses, divided into an intervention group (*n* = 52) and a control group (*n* = 47). The intervention was a mobile-based eight-week guided self-help mindfulness-based programme, delivered through WeChat, with five sessions per week using audio and text materials. The study reported significant reductions in occupational burnout, as measured by the MBI-HSS (Maslach Burnout Inventory—Human Services Survey), and substantial improvements in psychological resilience, assessed using the CD-RISC. The intervention was shown to be effective in both reducing burnout and enhancing resilience.

## 4. Discussion

Across the four studies, interventions achieved reductions in burnout, though the long-term sustainability of these improvements varied. For instance, Alenezi et al. [26] found that a two-day workshop temporarily reduced burnout, but the effects faded by six months, suggesting limited long-term impact. Similarly, Laker et al. [24] reported moderate reductions in burnout following an eight-week psycho-educational programme, though there was a slight decline in effect size at the six-month follow-up. While it is unclear whether the decline reflects natural fading or other factors, research shows that without ongoing support, early gains in resilience and accomplishment often diminish over time [28,29]. A meta-analysis by Cohen et al. [30] on healthcare worker burnout interventions reported that interventions lasting eight weeks or longer consistently yielded more sustained improvements, underlining the importance of intervention length for burnout prevention. These findings reinforce the current understanding that burnout interventions are most effective when supplemented by continuous support and regular sessions to sustain benefits over the long term.

Short-term interventions are more prevalent in the literature, potentially due to their lower cost, ease of implementation, and the immediate relief they offer. However, the transient nature of these improvements suggests a need to explore longer, more sustained interventions that can offer a lasting impact. Additionally, digital platforms, such as Wang et al.’s [25] mobile-based intervention, offered additional flexibility and scalability, especially valuable in high-demand environments like mental healthcare. However, it must be acknowledged that the success of digital interventions depends heavily on user engagement and adherence. Although Wang et al.’s [25] results showed that digital approaches can be effective, their success largely depended on keeping participants consistently engaged throughout the intervention [31], which can be challenging. Evidence from the digital mental health field supports this finding; for example, Gan et al. [32] found that engagement was significantly linked to positive outcomes in digital mental health interventions, especially when support tools like reminders and interactive content were included. Although not focused on mental health nurses, the study suggests that engagement-enhancing features could be adapted for high-stress nursing roles. In contrast, Wampole and Bressi’s [27] small pilot study demonstrated only marginal improvements, likely due to limited sample size and the absence of engagement-enhancing features, underscoring the importance of digital design and interactivity in maximising effectiveness.

An important theme that emerged was the role of participant engagement in the success of interventions. Studies with higher levels of engagement, such as the group-based psychoeducational programme in Laker et al. [24], tended to report more substantial and sustained reductions in burnout. Conversely, the limited engagement and small sample size in Wampole and Bressi [27] may have contributed to the intervention’s more limited success. This finding aligns with previous research conducted in hospital-based healthcare settings, showing that active participation is a strong predictor of intervention success [32,33]. Active engagement helps participants internalise coping strategies, enhance resilience, and apply learned skills in real-world scenarios [34]. By fostering ownership of their mental health, they are more likely to adopt these practices, leading to lasting behavioural changes [35]. Additionally, active participation boosts motivation and accountability, enhancing adherence to intervention protocols and effectiveness [36].

The limited four studies primarily took a reactive approach, aiming to reduce burnout symptoms after they had already manifested. While this provides symptomatic relief, a proactive approach that focuses on early intervention could minimise burnout’s onset by addressing its root causes, such as chronic stress, work overload, and insufficient support systems [37]. Proactive interventions might include regularly employed resilience training, stress management programmes, and systemic changes in the workplace to reduce high stress demands [11,38]. Burnout is an interconnected psychosocial issue that exists within a broader system of related factors [39]. Hence, effective intervention design must therefore adopt a systems view, addressing not only burnout itself but also its precursors and consequences [40]. Indeed, Edú-Valsania et al. [6] state that chronic stress, a known precursor to burnout, escalates over time, leading to emotional exhaustion, reduced productivity, and ultimately burnout. Burnout further impacts daily functioning, job satisfaction, and quality of life [11]. This cycle emphasises the need for interventions that address both the causes and effects of burnout [41].

The unique demands of mental health settings significantly shaped the effectiveness of interventions. It appeared that the inherent challenges of these environments directly impacted both the prevalence and intensity of burnout among nursing staff [42,43]. All four studies in this review, despite being limited, were conducted in specialised mental health facilities, such as psychiatric hospitals, where burnout is prevalent due to systemic factors like staffing shortages, high patient loads, and limited resources [44]. These conditions, as evidenced in Maglalang et al. [45], create a demanding work environment that intensifies both the emotional and physical strain on nurses, leading to exhaustion, reduced job satisfaction, and high turnover rates. Staffing shortages lead to increased workloads, resulting in excessive hours, missed breaks, and limited recovery time, which can drive stress to burnout [46]. Furthermore, high patient loads and complex cases increase the emotional toll, as nurses are tasked with managing frequent crises and providing continuous support to patients with severe mental health needs [47]. Limited resources further compound these pressures, as nurses may lack adequate tools, staffing support, or administrative backing, contributing to a sense of helplessness and frustration [43]. These challenges not only increase the risk of burnout but also negatively impact nurses’ job satisfaction and commitment, leading to higher turnover rates, a cycle that perpetuates staffing issues within mental health facilities [48]. Tailored interventions aimed at reducing emotional exhaustion, managing compassion fatigue, and enhancing coping strategies may be essential for reducing burnout effectively within these high-stress environments [49].

This systematic review included only four studies, each with unique strengths and limitations in design and quality. While numerous studies have explored burnout interventions in mental health settings, only four met the stringent inclusion criteria of using robust designs, such as RCTs or pre–post intervention models. The inclusion criteria aimed to enhance the validity and reliability of the findings by focusing on studies with rigorous methodologies; however, this also limited the number of studies available for review. We acknowledge that several other studies identified during the initial search, while excluded due to methodological limitations, still offer valuable contextual insights. These studies, which lacked control groups, used qualitative designs, or did not employ validated burnout measures, highlight emerging practices and challenges in addressing burnout among mental health nurses. While not included in the formal synthesis, they provide important background and underscore the broader interest and ongoing efforts in this area of research.

The quality of evidence across these studies varied, revealing both strengths and limitations in their findings. Alenezi et al. [26] and Laker et al. [24], which had larger sample sizes and employed quasi-experimental and RCT designs, respectively, offered more robust and potentially generalizable results. In contrast, Wampole and Bressi [27] faced challenges due to its small sample size and pilot nature, raising concerns about the generalizability of its findings for broader mental health nursing populations. However, all studies used validated burnout measures like the MBI and the OLBI, enhancing the credibility of the outcomes. Despite this, the risk of bias was a concern, especially in non-randomised studies such as Alenezi et al. [26], where lack of randomisation and blinding could lead to selection bias. While randomisation reduces bias, it can disrupt the relational dynamics important for peer support in burnout interventions. This highlights the need for study designs that balance methodological rigour with the benefits of interpersonal connection, particularly in high-stress healthcare settings. Wang et al. [25] also raised concerns about deviations from intended interventions and selective outcome reporting, which could affect result interpretation.

This review highlights a significant gap in data specific to burnout interventions for mental health nurses, indicating the need for focused research and approaches to address their unique stressors. Research that directly involves mental health nurses in the intervention design stage (e.g., surveys, focus groups, pilot sessions) could help tailor these strategies to their specific needs, making them more practical and effective. These approaches allow nurses to share insights about their daily stressors, preferred coping strategies, and practical constraints. By integrating their feedback into the intervention development process, the resulting strategies can better address their specific needs and challenges, making the interventions more relevant and applicable [50]. This collaborative design not only enhances the usability of interventions but also fosters a sense of ownership and engagement, increasing the likelihood that nurses will actively participate and benefit from the programmes.

The findings also underscore the importance of sustained interventions for meaningful burnout reduction. Short-term strategies, though beneficial for quick relief, may fail to maintain long-term impact and lead to repeated burnout cycles [46]. In contrast, long-term interventions, while potentially more resource-intensive, establish enduring support systems and coping mechanisms that help prevent burnout recurrence [30,51]. Therefore, healthcare organisations should prioritise continuous support, integrating approaches that balance accessibility with meaningful human engagement. While digital platforms can enhance reach, particularly in resource-limited settings, they should complement, rather than replace, interpersonal support, ensuring that compassionate, face-to-face interactions remain central to burnout prevention efforts in high-stress environments such as mental health wards.

Combining individual-focused strategies, such as stress management and resilience training, with broader organisational adjustments, like reducing workload and improving staffing levels, also provides a more comprehensive solution for tackling burnout [37]. This combined approach addresses both personal coping skills and systemic factors that contribute to burnout, creating a dual layer of support for mental health nurses [13]. Future research should examine the effectiveness of these strategies over both the short and long term, as this can reveal how quickly improvements appear and how well they hold up over time. By clarifying these immediate and lasting impacts, studies can help identify which combinations of individual and organisational interventions best support mental health nurses’ well-being and reduce burnout sustainably.

To strengthen the evidence base, future research should prioritise quasi-experimental designs, which include comparison groups and offer greater control over confounding variables. In contrast, pre-experimental single-group designs lack such controls, limiting causal inference and the ability to attribute outcomes to the intervention. Future studies should ideally involve more RCTs, specific to mental health nursing populations. RCT designs improve reliability, reduce bias, and provide clearer cause-and-effect insights [20,21]. While our review emphasises individual-level interventions, burnout in healthcare can be largely driven by systemic issues such as understaffing, administrative overload, and lack of managerial support. Addressing these requires structural reforms, like safe staffing policies, leadership development, and workflow redesign and policy-level action to create supportive environments. Hence, investigating a combination of individual and organisational interventions across diverse settings with nurses from various cultural backgrounds will help establish the generalisability of burnout strategies and identify any necessary adaptations for specific populations. Ultimately, tailoring these interventions to the distinct needs of mental health nurses can support both nurse well-being and patient care, fostering a healthier, more resilient mental health workforce.

### Limitations

A broad search strategy was applied across major electronic databases based on established inclusion criteria. However, some relevant studies may have been missed, especially those outside the criteria or in non-indexed journals. The specified date range might have excluded key studies, limiting the review’s comprehensiveness. Due to the heterogeneity of the included studies, a meta-analysis was not feasible; instead, a narrative synthesis guided by SWiM guidelines was employed. While this approach provides valuable insights, it lacks the statistical rigour of a meta-analysis. Only English-language studies were included, potentially omitting relevant research from non-English-speaking regions. While this review included studies from diverse cultural and healthcare settings, it is important to acknowledge that cultural, organisational, and systemic differences may influence both the experience of burnout and the effectiveness of interventions. For example, variations in nurse–patient ratios, societal attitudes toward mental health, and institutional support structures can shape how burnout manifests and how interventions are received. Therefore, while the findings offer valuable insights into potential strategies for reducing burnout among mental health nurses, caution must be exercised when generalising results across countries. Future research should explore culturally adapted interventions and examine how local context influences both implementation and outcomes to ensure relevance and effectiveness in diverse settings. Finally, despite the limited number and heterogeneity of available studies, a systematic review was chosen to ensure methodological transparency, minimise bias, and provide a reproducible synthesis. This approach strengthened the manuscript’s contribution by offering a structured and evidence-based overview that can inform both practice and future research.

## 5. Conclusions

Reducing burnout among mental health nurses is crucial for transforming healthcare environments. It strengthens workforce resilience, enhances patient care quality, promotes safety, and improves retention, all vital for sustaining healthcare systems. There is a pressing need for sustainable interventions specifically designed for mental health nurses, who are particularly vulnerable to burnout. While short-term solutions offer temporary relief, more comprehensive interventions and further research are essential to empower nurses and elevate standards in patient care.

## Figures and Tables

**Table 1:** Characteristics of the included studies.

Study Reference (Author, Year, Country)	Study Design	Objective	Sample Size	Setting (Hospital, Clinic, etc.)	Intervention Details (Type, Duration, Frequency)	Outcome Measures	Main Findings
Alenezi et al., 2019 [26] Saudi Arabia	Quasi-experimental study (non-equivalent pre-test and post-test design)	Measure the effects of a burnout prevention programme on mental health nurses working in Saudi Arabia	*n* = 296 Intervention Group: 154 Control Group: 142	Mental Health Hospital	Two-day (6 h per day) burnout prevention workshop	Maslach Burnout Inventory (MBI)	Significant reduction in burnout scores 1 month after the intervention; burnout scores increased by 6 months but did not return to baseline.
Wampole & Bressi, 2020 [27] USA	Quasi-experimental study (Pre–post intervention study)	Explore the effects of a mindfulness-based intervention to address burnout among inpatient psychiatric nurses	*n* = 5	Inpatient Psychiatric Facility	12-week social work-led mindfulness-based psychoeducational intervention, 1 h per week	Maslach Burnout Inventory-Human Services Survey (MBI-HSS)	Slight increase in Emotional Exhaustion (EE) from 2.9 to 3.2, Decrease in Depersonalisation (DP) from 2.0 to 1.7, and Personal Accomplishment (PA) remained consistent at 4.2
Laker et al., 2023 [24] UK	Pragmatic randomised controlled trial (pRCT)	Evaluate the effects of the Mind Management Skills for Life Programme on burnout and well-being among mental health nurses	*n* = 173 Immediate intervention group: 83 Delayed intervention group: 90	Mental Health Hospital	8-week group-based psychoeducational intervention (90 min sessions, 1 session per week)	Oldenburg Burnout Inventory (OLBI) Warwick-Edinburgh mental well-being scale (WEMWBS)	Moderate reduction in burnout (OLBI) and improved well-being (WEMWBS) after intervention (d = 0.60). Gains maintained at 6-month follow-up, though effect size reduced slightly.
Wang et al., 2024 [25] China	Randomised controlled trial (RCT)	Evaluate the effects of a guided self-help mindfulness intervention on psychological resilience and job burnout among psychiatric nurses	*n* = 118 Intervention Group: 52 Control Group: 47	Psychiatric Hospital	8-week guided self-help mindfulness-based intervention using audio and text materials via WeChat, with 5 sessions per week	MBI-HSS Five Facet Mindfulness Questionnaire (FFMQ) Connor–Davidson Resilience Scale (CD-RISC)	Significant reduction in occupational burnout and increase in psychological resilience in the intervention group compared to the control group.

## Data Availability

Data sharing is not applicable. No new data were created or analysed in this study.

## Appendix A

**Table A1:** Utilisation of PICO framework f or identifying research terms.

PICO Component	Main Concepts/Theoretical Constructs	Synonyms/Related Terms
(P) Population	Mental health nurses globally	(“psychiatric nurse * “OR” mental health nurse * “OR” community health nurse * “OR” behavioral health nurse * “OR” behavioural health nurse * “OR” psychiatric registered nurse * “OR” psychiatric-mental health nurse * “OR” community psychiatric nurse * “OR” psychiatric nurse practitioner * “OR” community mental health nurse *”)
(I) Intervention	Workplace interventions for burnout reduction	(“intervention * “OR” management “OR” program * “OR” strateg * “OR” measure * “OR” workshop * “OR” training * “OR” seminar * “OR” psychosocial intervention * “OR” burnout program * “OR” stress management “OR” wellness initiative * “OR” resilience training “OR” mindfulness programs “OR” Employee support programs “OR” Mental health support “OR” Workload management strategies “OR” Self-care strategies “OR” Peer support interventions “OR” Organizational policies for well-being”)
(C) Comparison	Pre-intervention status or absence of intervention	(“Usual care “OR” Standard practices “OR” No intervention “OR” Control group “OR” Alternative treatments “OR” Non-specific support “OR” Traditional workload management”)
(O) Outcome	Measures of burnout	(“Burnout scales “OR” Maslach Burnout Inventory scores “OR” Professional Quality of Life “OR” Job satisfaction levels “OR” Nurse retention rates “OR” Copenhagen Burnout Inventory “OR” Oldenburg Burnout Inventory “OR” Depression Anxiety Stress Scales (DASS) “OR” Stress symptoms “OR” Emotional Exhaustion Scale “OR” Shirom-Melamed Burnout Measure “OR” Occupational Fatigue Exhaustion Recovery (OFER) Scale “OR” Pediatric Burnout Inventory “OR” Burnout Clinical Subtype Questionnaire “OR” Work engagement”)

**Table A2:** Summary of inclusion and exclusion criteria.

Criteria	Inclusion Criteria	Exclusion Criteria
Population	Studies including mental health nurses actively working in healthcare settings globally. This includes psychiatric nurses, community health nurses, behavioural health nurses, etc.	Studies including non-mental health nursing staff or where mental health nurse data cannot be isolated.
Intervention	Studies evaluating specific workplace interventions aimed at reducing burnout among mental health nurses.	Studies focusing on general healthcare or hospital-wide interventions not specifically targeting burnout among mental health nurses.
Comparison	Studies with a control or comparison group such as usual care, no intervention, pre–post studies, or alternative interventions.	Studies lacking a control, pre-intervention, or comparison group.
Outcome	Studies measuring outcomes related to burnout using validated scales such as the Maslach Burnout Inventory (MBI), Copenhagen Burnout Inventory (CBI), and others, with pre- and post-intervention data.	Studies not measuring burnout or related mental health outcomes as primary or secondary endpoints, or studies without pre- and post-intervention measurements.
Study Design	Quantitative or mixed-methods empirical studies, including randomised controlled trials, cohort, cross-sectional, case–control, interventional, and observational studies.	Systematic reviews and meta-analyses, purely qualitative studies, case studies, narrative reviews, opinion pieces, and conference abstracts.
Geographic Focus	Studies conducted globally, without restriction to any specific country or region.	Studies that focus exclusively on regions or countries where the healthcare context or data do not apply to global mental health nursing practices (e.g., highly localised healthcare practices).
Timeframe	Studies published within the last 10 years.	Studies published more than 10 years ago.
Language	Studies that were published in English.	Studies that were not published in English.
Peer Review	Studies that have undergone peer review and are published in scientific journals.	Grey literature, unpublished theses/dissertations, and non-peer-reviewed articles.

**Table A3:** Completed risk of bias assessment table.

Study Reference (Author, Year)	Domain	Rating	Justification	Example from Study	Potential Bias	Potential Impact on Findings	Notes
Alenezi et al., 2019 [26]	Study Design	High Risk	Quasi-experimental design, with no randomisation process	Participants were not randomly assigned to intervention/control groups	Selection bias	Limits generalisability; potential confounding between groups	Non-randomised design introduces potential biases
	Deviations from intended interventions	Low Risk	Participants followed the intervention plan with minimal deviation	The two-day burnout prevention workshop was completed as planned	None	Reliable reflection of intervention effects	Intervention was delivered as intended
	Outcome measurement	Low Risk	Outcomes measured using validated tools (e.g., MBI)	Burnout measured using the Maslach Burnout Inventory (MBI)	None	High reliability in reported outcomes	Validated measurement tools used
	Missing outcome data	Low Risk	Minimal attrition reported, and missing data accounted for	All participants accounted for in final analysis	None	Reduces risk of attrition bias	Robust analysis with complete data
	Selection of reported results	Low Risk	All outcomes pre-specified in the protocol were reported	Burnout and psychological outcomes as listed in the protocol were included	None	Reliable reporting and transparent outcome measures	Full transparency in reported results
Wampole & Bressi, 2020 [27]	Study Design	High Risk	Pilot study with very small sample size (*n* = 5) and no control group	No randomisation and no control group	Selection bias	Limited generalisability and statistical power	Small sample size reduces confidence in conclusions
	Deviations from intended interventions	Low Risk	Participants adhered to the mindfulness-based intervention as planned	12-week mindfulness programme conducted as per protocol	None	Reliable assessment of intervention	Intervention delivered as planned
	Outcome measurement	Low Risk	Burnout measured using validated MBI-HSS tool	MBI-Human Services Survey was used to assess burnout	None	Reliable outcome measurement	Validated tool used
	Missing outcome data	Low Risk	No missing data reported for this small sample	Complete follow-up reported for all participants	None	No impact from missing data	Full data retention
	Selection of reported results	Low Risk	All pre-specified outcomes were reported	Burnout scores and mindfulness outcomes reported as intended	None	Transparent outcome reporting	Limited due to small sample size
Laker et al., 2023 [24]	Study Design	Low Risk	Randomised controlled trial with two groups (immediate vs. delayed intervention)	Participants randomly assigned to immediate or delayed intervention groups	None	High internal validity and generalisability	Strong design supports findings
	Deviations from intended interventions	Low Risk	Intervention was delivered as planned, with minimal deviations	Participants attended eight weekly sessions as intended	None	Reliable intervention assessment	Protocol followed as planned
	Outcome measurement	Low Risk	Outcomes measured using validated tools (e.g., OLBI and WEMWBS)	Burnout and well-being measured with validated tools	None	High reliability of measured outcomes	Well-validated tools used
	Missing outcome data	Low Risk	Minimal attrition reported, and missing data was accounted for appropriately	Low dropout rate, and all data included in analysis	None	No significant bias due to missing data	Robust data handling
	Selection of reported results	Low Risk	All pre-specified outcomes were reported	Burnout and well-being outcomes fully reported	None	No selective reporting bias	Transparent and comprehensive reporting
Wang et al., 2024 [25]	Study Design	Low Risk	Randomised controlled trial with intervention and control groups	Participants randomly assigned to intervention or control groups	None	High internal validity	Strong randomisation ensures robust findings
	Deviations from intended interventions	Some Concerns	Some participants deviated from the intervention by not completing all five weekly sessions	Inconsistent adherence to the WeChat-delivered mindfulness programme	Performance bias	May impact consistency of intervention effects	Adherence issues could weaken effect size
	Outcome measurement	Low Risk	Outcomes measured using validated MBI-HSS and CD-RISC tools	Burnout and resilience measured using well-established tools	None	Reliable and validated outcome measures	Strong outcome measurement tools used
	Missing outcome data	Low Risk	Minimal missing data; follow-up accounted for in the final analysis	Small percentage of participants lost to follow-up	None	No significant bias due to missing data	Robust data analysis
	Selection of reported results	Low Risk	All pre-specified outcomes were reported	Burnout, resilience, and mindfulness outcomes fully reported	None	Transparent outcome reporting	No significant reporting bias

**Table A4:** Completed critical appraisal table.

Study Reference (Author, Year)	Study Design	Sample Size	Intervention Type	Quality Rating	Risk of Bias	Key Findings	Comments
Alenezi et al., 2019 [26]	Quasi-experimental	*n* = 296	Two-day burnout prevention workshop	Moderate	High selection and confounding bias due to lack of randomisation	Significant reduction in burnout one month post-intervention, but effects diminished by six months.	Lacked randomisation; long-term sustainability of intervention effects is questionable.
Wampole & Bressi, 2020 [27]	Quasi-experimental (pilot)	*n* = 5	12-week mindfulness-based psychoeducational intervention	Low	High risk of selection bias due to small sample size and non-random allocation	Mixed results: depersonalisation improved, emotional exhaustion worsened, personal accomplishment unchanged (3 measured dimensions of burnout)	Small sample size significantly limits generalisability; pilot nature impacts strength of conclusions.
Laker et al., 2023 [24]	Pragmatic RCT	*n* = 173	Eight-week group-based psychoeducational intervention	High	Low risk of bias overall	Moderate reductions in burnout and improvements in well-being, with slight attenuation at six-month follow-up	Well-conducted RCT with strong methodology; robust randomisation and adequate follow-up data.
Wang et al., 2024 [25]	RCT	*n* = 118	Eight-week guided self-help mindfulness intervention	High	Low risk of bias overall	Significant reductions in burnout and increases in psychological resilience	Digital delivery via mobile platforms offers scalable potential, but slightly inconsistent follow-up data.
